# Renal disease in critical care patients

**DOI:** 10.1186/2197-425X-3-S1-A266

**Published:** 2015-10-01

**Authors:** M Herrera-Gutierrez, E Banderas-Bravo, D Arias-Verdú, J Barrueco-Francioni, G Quesada-García, G Seller-Pérez

**Affiliations:** Complejo Universitario Carlos Haya, Malaga, Spain; Complejo Universitario Carlos Haya, ICU, Malaga, Spain

## Introduction

Recently a new scenario has been proposed for renal dysfunction with the coining of the term “renal disease” (RD) as a whole, integrating the classic terms of CKD, the no so classic AKI and the emergent AoC renal disease.

## Objectives

To define the epidemiology of this entity in ICU and its prognosis at hospital discharge.

## Methods

Post-hoc analysis of a prospective observational cohort from a previous study on AKI conducted in our Unit. We detected previous renal disease as stated in the medical records, and AKI or AoC based in KDIGO criteria (any degree). We performed a Cox proportional risks survival analysis. Data are shown as mean (SD) and Hazard Ratio (95% CI).

## Results

279 patients aged 54,3(18,4) years, 69,5% men, admission APACHE II 29,8(10,3), basal creatinine 1,04(0,79) and higher creatinine 1,75(7,36) in day 3(1-9). Our population is close to the usual ICU case-mix (22,9% trauma, 16,1% cardiac surgery, 11,5% sepsis, 9,3% urgent surgery, 5,4% elective surgery, 8,6% trasplant y 26,2% other). 20,1% reported diabetes y 35,5% high blood pressure. 46,2% needed vasopressors y 36,9% had infection.

In our cohorts, 43% developed AKI during hospital stay, 5,7% AoC and 15,4% had chronic disease. Only 35,9% of our cases did not have RD.

Hospital mortality was 45,8% for DRA, 25% for AoC, 6,3% for chronic disease and 6% for those cases without RD

## Conclusions

Renal disease shows a high incidence for ICU patients, but its repercussion on hospital mortality is related to the stage of disease, being highly relevant when a new injury is detected in a patient with a previous normal renal function and less so when it happens in a patient with a previous history of renal disease.

**Figure 1:**
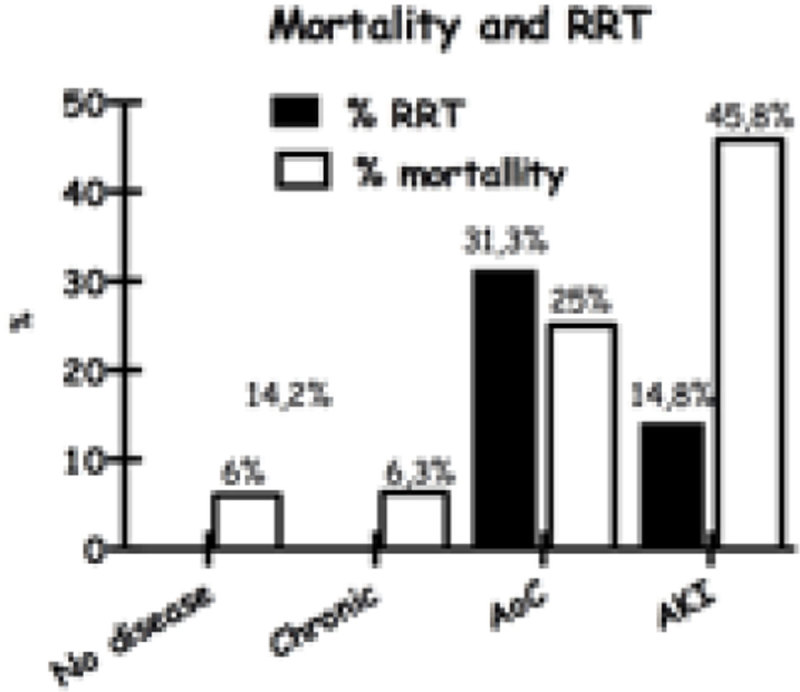
**In the multivariate analysis, APACHE, age, vasopressors, sepsis and RD (specifically AKI and AoC) were related to mortality, with a hazard ratio of 4,49 (IC 1,71-11,7) for AKI and 2,44 (IC 0,63-9,39) for AoC**

**Figure:**
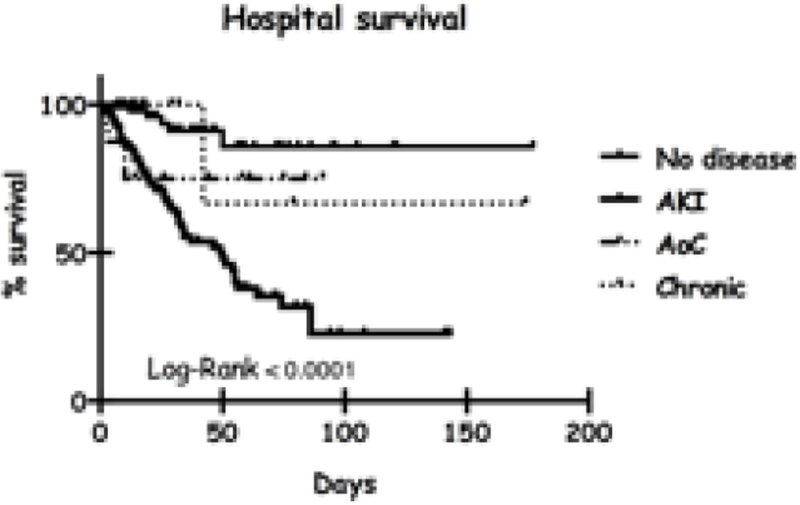
Figure 2

